# The neuro-steroid, 3*β* androstene 17*α* diol exhibits potent cytotoxic effects on human malignant glioma and lymphoma cells through different programmed cell death pathways

**DOI:** 10.1038/sj.bjc.6603894

**Published:** 2007-07-17

**Authors:** M R Graf, W Jia, R M Loria

**Affiliations:** 1 Department of Neurosurgery and the Massey Cancer Center, Virginia Commonwealth University Medical Center, PO Box 980631, Richmond, VA 23298-0631, USA; 2 Departments of Microbiology and Immunology and Pathology, Virginia Commonwealth University Medical Center, PO Box 980631, Richmond, VA 23298-0631, USA

**Keywords:** androstene neuro-steroids, glioblastoma, lymphoma, malignant glioma, programmed cell death

## Abstract

The neuro-steroids 3*β*-androstene-17*α*-diol (17*α*-AED), 3*β*-androstene-17*β*-diol (17*β*-AED), 3*β*-androstene-7*α*,-17*β*-triol (7*α*-AET) and 3*β*-androstene-7*β*,-17*β*-triol (7*β*-AET) are metabolites of dehydroepiandrosterone and are produced in neuro-ectodermal tissue. Both epimers of androstenediols (17*α*-AED and 17*β*-AED) and androstenetriols (7*α*-AET and 7*β*-AET) have markedly different biological functions of their chemical analogue. We investigated the cytotoxic activity of these neuro-steroids on human T98G and U251MG glioblastoma and U937 lymphoma cells. Proliferation studies showed that 17*α*-AED is the most potent inhibitor, with an IC_50_ ∼15 *μ*M. For T98G glioma, 90% inhibition was achieved with 25 *μ*M of 17*α*-AED. Other neuro-steroids tested only marginally suppressed cell proliferation. Reduced cell adherence and viability could be detected after 18 h of 17*α*-AED exposure. Treatment with 17*α*-AED induced a significant level of apoptosis in U937 lymphoma cells, but not in the glioma cells. Cytopathology of 17*α*-AED-treated T98G cells revealed the presence of multiple cytoplasmic vacuoles. Acridine orange staining demonstrated the formation of acidic vesicular organelles in 17*α*-AED-treated T98G and U251MG, which was inhibited by bafilomycin A1. These findings indicate that 17*α*-AED bears the most potent cytotoxic activity of the neuro-steroids tested, and the effectiveness may depend on the number of hydroxyls and their position on the androstene molecule. These cytotoxic effects may utilize a non-apoptotic pathway in malignant glioma cells.

Glioblastomas are grade IV astrocytic neoplasms derived from the glial lineage and represent ∼50% of primary central nervous system (CNS) tumours. They are essentially incurable with a median survival of approximately 18 months after primary diagnosis, regardless of treatment. Traditional treatment for this disease is surgical resection accompanied by radiation and is generally considered palliative with the 5-year survival rate of 2% (Surawicz *et al*, 1998).

Nitrosourea chemotherapeutic agents are often used as adjuvant with radiation; however, the therapeutic benefit of the inclusion of these agents is controversial (Walker *et al*, 1980; Stupp and Weber, 2005a, 2005b). The reduced efficacy of these compounds may be attributed to their limited penetration through the blood brain barrier, and the resistance of migrating glioblastoma cells to apoptosis (Kemper *et al*, 2004; Lefranc *et al*, 2005). Temozolomide is an alkylating agent that can access the CNS, and recent clinical studies have showed that temozolomide administration combined with radiation therapy can improve the overall survival of malignant glioma patients (Stupp *et al*, 2001; Stupp and Weber, 2005a, 2005b). Stupp and Weber (2005a, 2005b) demonstrated that temozolomide combined with radiation therapy prolonged the survival of glioblastoma patients with a median increase in survival of 2.5 months. Despite the recent advancement in the use of temozolomide in the treatment of glioblastomas, the prognosis of these patients remains poor and there continues to be a need for the development of effective therapies for the treatment of this disease.

Androstene steroids are produced in neuro-ectodermal tissue by the metabolism of the common steroid dehydroepiandrosterone (DHEA) (Lathe, 2002). The androstenes are neuro-steroids that exist in *α*- and *β*-epimeric forms (Figure 1) and, although chemically identical, the biological actions of the two epimers are distinctly different. In this regard, researchers have demonstrated that 3*β* androstene 17*α* diol (17*α*-AED) can inhibit the proliferation and induce apoptosis of human U937 and HL60 lymphoma cells (Huynh and Loria, 1997; Loria, 2002). In addition, human MDA-MB231 and ZR75-1 breast cancer cells also undergo apoptotic cell death when treated with 17*α*-AED by a mechanism that is independent of oestrogen and androgen receptors (Huynh *et al*, 2000). 17*α*-AED does not have inhibitory or apoptotic effects on normal cells (Loria, 2002). In contrast, the epimer, 3*β* androstene 17*β* diol (17*β*-AED is an enhancer of immune regulation (Loria, 2002). In this regard, it has been shown that 17*β*-AED can increase host resistance to lethal infections and acute radiation poisoning; enhance TH1 cytokine production by immune cells and counteract hydrocortisone induced immune-suppression (Padgett and Loria, 1994; Loria, 1997; Ben Nathan *et al*, 1999; Loria *et al*, 2000; Padgett *et al*, 2000). Furthermore, a metabolite of 17*β*-AED, 3*β* androstene 7*β*, 17*β* triol (7*β*-AET), which has a third hydroxyl group in the *β*-position on the seventh carbon atom, is significantly more immuno-potent than 17*β*-AED (Loria, 2002).

In the current study, we evaluated the cytotoxic properties of these four androstene neuro-steroids on human T98G and U251MG malignant glioma cell lines. We have reported that 17*α*-AED, but not 17*β*-AED, is inhibitory to human U937 lymphoma cells (Huynh and Loria, 1997); however, this is the first comparative investigation of the cytotoxic potential of 7*α*-AET and 7*β*-AET. This report shows that, among these androstenes, 17*α*-AED is the most potent inhibitor of human glioma and lymphoma cell proliferation. However, the cytotoxic effects of 17*α*-AED on malignant glioma cells as compared to lymphoma cells appear to be mediated by different pathways. In this regard, 17*α*-AED induces apoptosis in lymphoma cells while treated glioma cells appear to undergo an autophagic form of cell death. This is the first report of the antiglioma effects of androstene neuro-steroids.

## MATERIALS AND METHODS

### Cell lines and culture

Glioma cells were cultured in DMEM supplemented with 10% FBS and non-essential amino acids as adherent monolayers at 37°C, passed biweekly with trypsin in the absence of antibiotics. Cell lines were routinely screened for mycoplasma contamination (MycoTect, Invitrogen, Carlsbad, CA, USA). All tissue culture reagents and supplements were obtained from Invitrogen. The human glioblastoma cell lines T98G and U251MG were maintained by the Neuro-Oncology Research Group at Virginia Commonwealth Medical Center (Richmond, VA, USA). The T98G and was originally acquired from the American Type Culture Collection (ATCC, Manasas, VA, USA), and the U251MG cell line was obtained from the European Collection of Cell Cultures (Wiltshire, UK). The human U937 histiocytic lymphoma cell line (Sundstrom and Nilsson, 1976) was obtained from the ATCC and grows in suspension.

### Proliferation assay

The proliferation of tumour cells was assessed by the incorporation of ^3^H-TdR (Amersham Biosciences, Piscataway, NJ, USA) in a 96-well, flat-bottom tissue culture plate. T98G and U251MG glioma cells (1 × 10^4^ well^−1^) and U937 lymphoma cells (2 × 10^4^ well^−1^) were cultured in the presence of 17*α*-AED, 17*β*-AED, 7*α*-AET or 7*β*-AET (provided by Dr Loria) at concentrations ranging from 200–3 *μ*M in a final volume of 200 *μ*l well^−1^ for 3 days. Control wells were treated with an equivalent amount of vehicle (50% PEG400/50% ethanol). During the last 15 h of culture, cells were pulsed with 1 *μ*Ci of ^3^H-TdR and stored at −80°C. Incorporation of ^3^H-TdR was used as a measure of proliferation and was analysed using a 96-well plate harvester and a beta-plate reader (Packard, Meridien, CT, USA). Data are expressed as percent reduction in proliferation of the mean of triplicate experimental cultures as compared to the mean of sham-treated cultures.

### Cell viability assay

A total of 5 × 10^5^ T98G or U251MG glioma cells were seeded in a six-well cluster plate and incubated for 6–8 h, after which medium was removed and wells were rinsed with Hank's Balanced Salt Solution (HBSS, Invitrogen). Fresh medium supplemented with 17*α*-AED or equivalent amount of vehicle was added to the cultures at a concentration of 10 *μ*M for T98G cells and 15 *μ*M for U251MG cells. After 18 h of exposure to 17*α*-AED, cultures were viewed with a phase-contrast microscope and photographed. Floating cells were collected. Adherent cells were trypsinized, pooled with the non-adherent cells, centrifuged and the pellet was resuspended in phosphate-buffered saline (PBS). U937 cells were seeded at a concentration of 4 × 10^5^ cells ml^−1^ and after 6–8 h, 17*α*-AED (10 *μ*M final) or vehicle was added to the cultures. After 18 h, cells were collected, pelleted and resuspended in PBS. Over 200 cells in total were counted in each sample on a hemacytometer, and viable live cells were distinguished from dead cells by trypan blue exclusion. Mean values s.e. are shown.

### Assay for DNA fragmentation

Tumour cells were seeded at a concentration of 1 × 10^5^ cells well^−1^ in a six-well cluster plate and incubated for 6–8 h before addition of 17*α*-AED (10 *μ*M for T98G and U937; 15 *μ*M for U251MG) or equivalent amount of vehicle. After 4 days, detached and adherent cells were collected as previously described and washed twice with PBS. Terminal deoxynucleotidyltransferase dUTP nick end labelling (TUNEL) was used for the detections of DNA cleavage in the treated cells (APO-DIRECT, BD Biosciences, San Diego, CA, USA). Cells were analysed on a FACSCanto flow cytometer (BD Biosciences). Forward scatter threshold was adjusted to omit cellular debris; 10 000 events were counted and positive TUNEL staining was read in the FL1 channel (FITC).

### Western blotting

Tumour cells were seeded in a six-well cluster plate and incubated with 17*α*-AED (10 *μ*M, final) or vehicle as described above. After 3 days, spent medium was collected, centrifuged and the pellet was added back to the original cell cultures. Cells were lysed with RIPA buffer, lysates were clarified by centrifugation and stored at −80°C. The protein concentration of the lysates was determined using a micro BCA protein assay kit (Pierce Chemicals, Rockford, IL, USA). A 25 *μ*g weight of total protein was run on a 4–12% NuPAGE MES gel (Invitrogen) and transferred to nitrocellulose membranes. Membranes were blocked with 5% non-fat dry milk+0.1% Tween-20 in PBS for 1 h. The membranes were incubated overnight with rabbit polyclonal Ab specific for poly (ADP-ribose) polymerase (PARP) or caspase 3 (both from Cell Signaling Technology, Beverly, MA, USA) at 4°C, washed with 0.1% Tween-20 in PBS and incubated with goat anti-rabbit Ab conjugated to horseradish peroxidase (Rockland Immunochemicals, Gilbertsville, PA, USA) for 1 h. Immunoreactive bands were visualized by chemiluminescence (SuperSignal, Pierce Chemicals). The same membranes were reblotted with a mouse anti-*β*-actin mAb (Sigma, St Louis, MO, USA), as described above, and used as a loading control.

### Cytopathology

For microscopic analysis, 5 × 10^4^ T98G glioma cells were added to each chamber of a four-chamber glass slide and incubated for 15 h. Medium was then removed and wells were gently washed with HBSS. Fresh medium was added and supplemented with 17*α*-AED for a final concentration of 10 *μ*M. After 30 h, medium was removed, wells were gently rinsed with HBSS, cultures were fixed with 5% buffered formalin in DMEM. Chamber slides were then subjected to routine haematoxylin and eosin staining. Cells were viewed with a Nikon Eclipse E800 microscope and images obtained with a Diagnostic Instruments Spot RT CCD camera.

### Detection of acidic vesicular organelles

Acidic vesicular organelles (AVO) can be detected in cells by staining with acridine orange (Traganos and Darzynkiewicz, 1994; Paglin *et al*, 2001). Tumours cells were seeded into six-well cluster plates and cultured in the presence of vehicle or 10–15 *μ*M 17*α*-AED as described above, for 3 days. In some cases, bafilomycin A1 (50 nM, Sigma) was added to the cultures for the last 48 h. At the end of the culture period, acridine orange (Sigma) was added to each culture (1.0 *μ*g ml^−1^ final) and cells were incubated for 15 min. Total floating and adherent cells were collected; washed with PBS and analysed on a FACSCanto flow cytometer. When excited with a 488 nm laser, the nucleolus of acridine orange stained cells fluoresces bright green which can be detected in the FL1 channel and acidic vesicles emit a bright red fluorescence which can be detected in the FL3 channel (Paglin *et al*, 2001; Kanzawa *et al*, 2003b). Forward scatter threshold was adjusted to omit cellular debris and 10 000 ungated events were analysed. Tumour cells containing AVO were identified as double positive cells in the Q2 quadrant of the FL1, FL3 histogram.

### Statistiscs

Statistical analysis was performed using the Student's *t*-test. Differences were considered to be significant when the calculated *P*-value was less than 0.05.

## RESULTS

### Androstene steroids inhibit tumour cell growth and induce cell death

**Figure 2:**
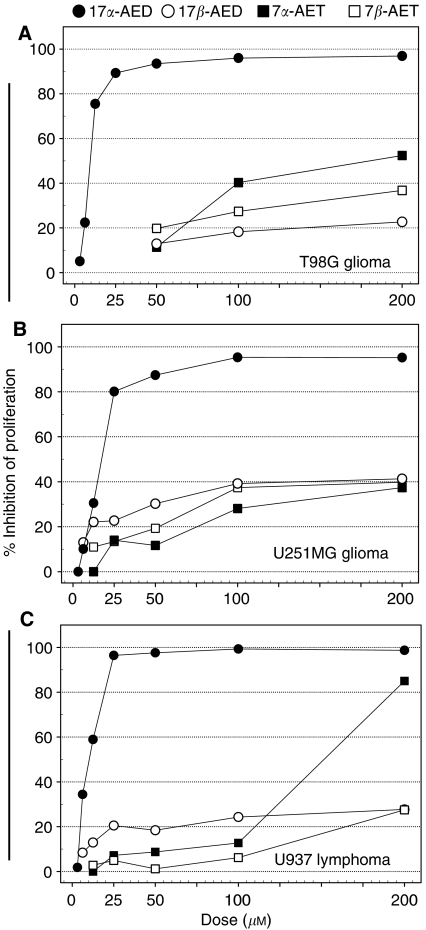
17*α*-AED is a potent inhibitor of tumour cell proliferation. (**A**) T98G glioma cells, (**B**) U251MG glioma cells and (**C**) U937 lymphoma cells were cultured for 3 days with titrated doses of 17*α*-AED (•); 17*β*-AED (○); 7*α*-AET (▪) or 7*β*-AET (□), in a standard proliferation assay. Incorporation of ^3^H-TdR was used as a measure of cell proliferation, and the percentage in which proliferation was inhibited in the androstene neuro-steroid-treated cultures as compared to sham-treated cultures is indicated. A representative experiment of at least two independent experiments is shown.

In order to determine the antiproliferative potential of the androstene neuro-steroids, human T98G glioma, U251MG glioma and U937 lymphoma cells were cultured in the presence of 17*α*-AED, 17*β*-AED, 7*α*-AET and 7*β*-AET at titrated doses ranging from 3–200 *μ*M, for 3 days. The results are shown in Figure 2, and demonstrate that 17*α*-AED is the most potent inhibitor of these neuro-steroids. In this regard, 17*α*-AED at a dose of 25 *μ*M inhibited the proliferation of T98G glioma and U937 lymphoma cells by 90% or greater and U251MG glioma cell proliferation was inhibited by 80%. For each tumour, there was a significant dose response to 17*α*-AED when used between 3 to 25 *μ*M. The approximate concentration of 17*α*-AED to inhibit 50% of cell proliferation (IC_50_) was 10 *μ*M for T98G and U937 and 15 *μ*M for U251MG. In contrast, 17*β*-AED, 7*α*-AET and 7*β*-AET could only suppress 30% or less of tumour cell proliferation at the 50 *μ*M dose, and these antiproliferative effects modestly increased at higher concentrations as compared to the effects of 17*α*-AED. Therefore, 17*α*-AED is significantly more effective in inhibiting malignant glioma and lymphoma cell proliferation than its chemically identical *β*-epimer (17*β*-AED); consequentially, nonspecificity does not appear to be an issue. Additionally, 17*α*-AED is also markedly more effective than either 7*α*-AET or 7*β*-AED, and the inhibitory effect of 17*α*-AED is dose dependent. Subsequent studies focused on the cytotoxic effects of 17*α*-AED when used at the approximate IC_50_ concentration.

**Figure 3:**
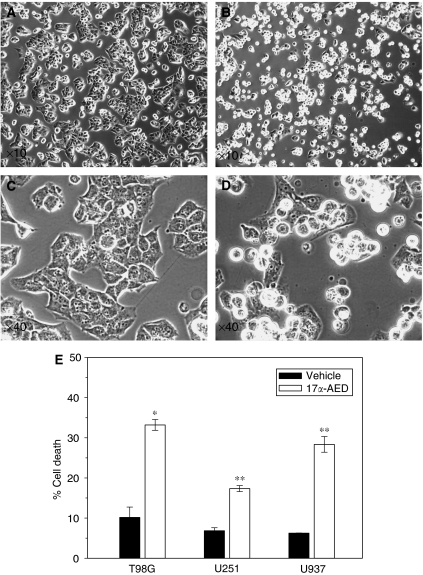
Cytotoxic effects of 17*α*-AED. Tumour cells were seeded onto culture dishes and incubated for ∼8 h before the addition of 17*α*-AED. After 18 h of treatment, cultures were photographed and the viability of cells was determined. (**A**, **C**) T98G cells treated with vehicle or (**B**, **D**) 10 *μ*M 17*α*-AED. (**E**) Cell death of T98G glioma and U937 lymphoma cells treated with 10 *μ*M 17*α*-AED and U251MG glioma cells treated with 15 *μ*M 17*α*-AED. Mean values of at least two independent experiments are shown. ^*^P=0.001, ^**^P⩽0.01.

In the proliferation studies above, it may be possible that 17*α*-AED interferes with the ability of the glioma cells to efficiently adhere to the tissue culture plate and form a healthy monolayer. To address this, we incubated the glioma cells for 6–8 h before adding 17*α*-AED to the cultures. This time period allowed the glioma cells to firmly attach to the tissue culture plates. Vehicle or 17*α*-AED (10 *μ*M) was then added to T98G cultures. The morphological effects of the neuro-steroid on the glioma culture could be detected as early as 8 h post-treatment, and included reduced cellular adherence and de-attachment of cells from the tissue culture plate. Figure 3A–D show phase-contrast images of a representative T98G culture after 18 h of exposure to vehicle or 17*α*-AED. The effect of 17*α*-AED (15 *μ*M) on U251MG glioma cultures was similar (data not shown). Immediately following photography, the viability of the cultures was assessed by trypan blue exclusion. The results, shown in Figure 3E, indicate that 17*α*-AED-treated T98G, U251MG and U937 cultures contain a significant percentage (33.2, 17.4 and 28.4%, respectively, *P*⩽0.01) of non-viable cells as compared to sham-treated cultures which was ⩽10%. Cell death in 17*α*-AED-treated T98G glioma and U937 lymphoma was approximately three times greater than of vehicle-treated cells.

### 17*α*-AED induces apoptosis of human U937 lymphoma cells, but not T98G and U251MG glioma cells

**Figure 4:**
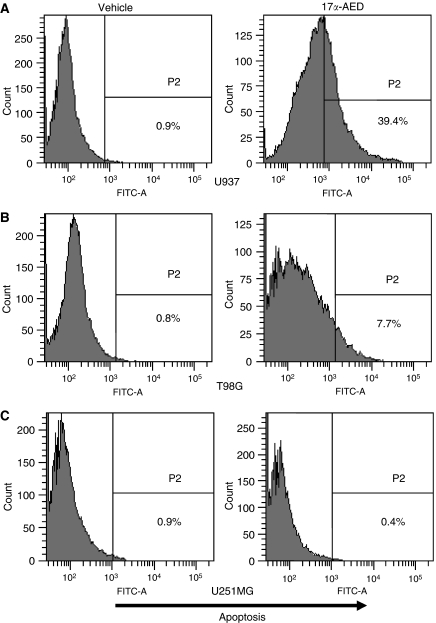
17*α*-AED treatment induces DNA fragmentation of U937 lymphoma cells. (**A**) U937 lymphoma cells; (**B**) T98G glioma cells and (**C**) U251MG glioma cells were treated with vehicle (left panels) or 17*α*-AED (right panels). After 4 days, the degree of DNA cleavage was assessed by TUNEL staining and used as a measure of apoptosis. Annotated numbers reflect the percentage of cells staining positive for apoptosis (*x*-axis, FITC-A). This experiment was performed twice and yielded similar results.

**Figure 5:**
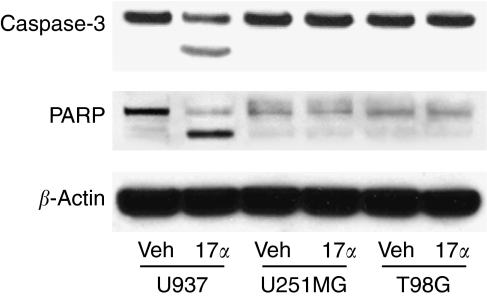
Treatment with 17*α*-AED induces cleavage of caspase 3 and PARP in U937 lymphoma cells, but not in T98G and U251MG glioma cells. Whole-cell lysates were obtained from human U937, T98G and U251MG cells treated for 3 days with vehicle or 17*α*-AED (10 *μ*M). A 30 *μ*g weight of total protein from these cells were subjected to Western blot analysis, and probed with an Ab specific for the total caspase 3 or PARP. A reduced level of pro-caspase 3 or PARP, accompanied by the presence of their smaller cleavage products can be seen 17*α*-AED-treated U937 cells as compared to cells treated with vehicle, but not in treated malignant glioma cells. An anti-*β*-actin mAb was used to show equal protein loading.

Several standard assays for apoptosis were conducted on U937 lymphoma and the malignant glioma T98G and U251MG cells treated with 17*α*-AED or vehicle for 3–4 days. Nuclear DNA fragmentation is a morphological change that occurs in apoptotic cells and is a classical feature of apoptosis (Nagata *et al*, 2003). A TUNEL assay was performed on the tumour cells treated with 17*α*-AED to detect DNA cleavage, which was quantified by flow cytometry. The results are shown in Figure 4. In the U937 lymphoma cultures treated with 17*α*-AED, 39.4% of the cells were TUNEL positive, indicating that these cells were apoptotic. Furthermore, induction of apoptosis by 17*α*-AED treatment of U937 lymphoma cells was dose dependent, as revealed by annexing V staining, with an LD_50_ between 6.25 and 12.5 *μ*M (data not shown). In contrast to U937 cells, only a low level, 7.7% of T98G glioma cells treated with 17*α*-AED were positive for TUNEL and the percentage of TUNEL-positive U251MG cells exposed to 17*α*-AED remained at the level of sham-treated U251MG cells. Proteolytic processing of the effector caspase 3 and PARP are biochemical events of apoptosis and the presence of their cleavage products are markers of this cell death pathway (Nunez *et al*, 1998; Oliver *et al*, 1998). Western blotting was conducted using whole-cell lysates from human U937, T98G and U251MG cells treated with 17*α*-AED, to determine the extent of caspase 3 and PARP cleavage. The results, shown in Figure 5, demonstrate that U937 lymphoma cells treated with 17*α*-AED have an increased level of cleaved caspase 3 and PARP as compared to cells treated with vehicle. In contrast, no cleavage of caspase 3 was detected in treated T98G and U251MG malignant glioma cells, and there was no change in background levels of PARP cleavage noted in the 17*α*-AED-treated glioma cells as compared to glioma cells exposed to vehicle. Caspase 3 cleavage and PARP processing was not detected in U937, T98G and U251MG tumour cells exposed to 17*α*-AED for 24 or 48 h, thereby excluding the possibility of early apoptotic cell death in these experiments (data not shown). These results demonstrate that 17*α*-AED treatment induces U937 lymphoma cells to undergo apoptosis, which is in accordance with our previous report (Huynh and Loria, 1997). However, the major cytotoxic effects of 17*α*-AED on malignant glioma cells appear to be mediated by a non-apoptotic pathway.

### Cytopathic effects of 17*α*-AED on T9G8 glioma cells

**Figure 6:**
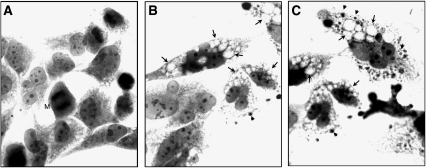
Cytopathology of T98G cells exposed to vehicle or 17*α*-AED (10 *μ*M) for 30 h. (**A**) T98G cultures treated with vehicle are composed of cells with epithelioid morphology; a large, pale staining nucleus with prominent nucleoli. A T98G cell undergoing mitosis (M). (**B**, **C**) Numerous cells in the 17*α*-AED-treated cultures show the presence of multiple cytoplasmic vacuoles (arrows) and small, basophilic bodies (arrow heads). Haematoxylin and eosin staining, × 100 magnification with oil.

Human T98G glioma cells were cultured for 30 h in the presence of vehicle or 17*α*-AED at a final concentration of 10 *μ*M. Analysis of haematoxylin and eosin-stained cultures revealed significant morphological alterations as a result of 17*α*-AED treatment. In the sham-treated T98G culture (Figure 6A), glioma cells displayed a large, pale staining nucleus containing prominent nucleoli, and were epithelioid in appearance. Also in the control culture, numerous mitotic figures were apparent and, in a small percentage of cells, marginal cytoplasmic vacuolization could be noted. In the 17*α*-AED-treated cultures (Figures 6B and C), very few mitotic figures were present, and a substantial number of T98G cells contained multiple, cytoplasmic vacuoles which, in some cases, appeared to displace the nucleus. Vacuolization was often accompanied by the presence of small, basophilic bodies. The presence of these vacuoles in the 17*α*-AED-treated T98G cells was also detected by flow cytometry as an increase of cellular side scatter (data not shown).

### 17*α*-AED induces autophagosome formation in malignant glioma cells

**Figure 7:**
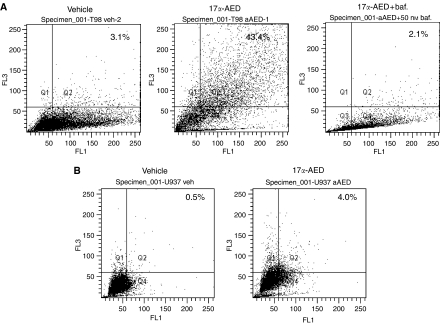
Acridine orange staining of 17*α*-AED-treated tumour cells indicates a high level of acidic vacuoles in T98G glioma cells, but not in U937 lymphoma cells. (**A**) Treatment with 10 *μ*M of 17*α*-AED (middle) induces a high level of AVO formation in T98G cells as compared to T98G cells treated with vehicle (left). The presence of bafilomycin A1 (baf., 50 nM) inhibits 17*α*-AED (10 *μ*M)-induced AVO formation in T98G cells (right). (**B**) A low level of acidic vacuoles can be detected in U937 cells treated with 10 *μ*M 17*α*-AED (right) as compared to sham-treated cells (left). Annotated numbers indicate the percentage of cells positive for acidic vacuoles, quadrant Q2. Representative results of at least three independent experiments are shown.

Cytoplasmic vacuolization is a feature of certain death pathways such as necrosis and autophagy, but not apoptosis, and the presence of acidic vacuoles or AVO is a characteristic of autophagic cell death (Paglin *et al*, 2001; Lefranc and Kiss, 2006). Vital staining with acridine orange was used to detect the presence of AVO in tumour cells treated with 17*α*-AED. The results shown in Figure 7A indicate that more than 40% of T98G glioma cells and only 4.0% of U937 lymphoma cells (Figure 7B) treated with the neuro-steroid contained AVO. A high percentage of AVO was also detected in U251 malignant glioma cells treated with 17*α*-AED, as compared to sham-treated cells (data not shown). Bafilomycin A1 specifically inhibits AVO formation by blocking the fusion of autophagosomes and lysosomes (Yamamoto *et al*, 1998). The addition of bafilomycin A1 prevented the generation of AVO in 17*α*-AED-treated T98G glioma cells (Figure 7A) and U251MG glioma cells (data not shown). These results suggest that 17*α*-AED induces autophagic cell death in human malignant glioma cells, but not in lymphoma cells.

## DISCUSSION

The anticancer effects of DHEA have been widely investigated and researchers have shown that treatment with DHEA can inhibit the growth of different tumours such as colon adenocarcinoma (Schulz *et al*, 1992), pancreatic carcinoma (Melvin *et al*, 1997) and breast cancer (Boccuzzi *et al*, 1993). Additionally, DHEA treatment has been shown to retard the onset of chemically induced cancers such as mammary, liver and prostate carcinogenesis in experimental rat models (Simile *et al*, 1995; Lubet *et al*, 1998; Rao *et al*, 1999). More recently, Yoshida *et al* (2003) reported that DHEA and its metabolites have antiproliferative effects on human hepatoma and human colonic adenocarcinoma cell lines. The androstene steroid molecules used in this study are naturally synthesized in neuro-ectodermal tissue by the metabolism of DHEA, and represent a true class of neuro-steroids. In this investigation, we examined the cytotoxic effects of chemically identical or similar androstene derivatives on two human malignant glioma cell lines and a human lymphoma cell line. Our findings demonstrate that the androstene neuro-steroids can inhibit the proliferation of these tumour cells, induce cell death, and, in the case of glioma cells, evoke cytopathological changes consistent with autophagy.

The results of the proliferation studies showed that there is a significant difference in the potency of the four androstene neuro-steroids. The percentage of growth inhibition for 17*α*-AED at 50 *μ*M dose was well above 80% for all of the tumours, clearly indicating that 17*α*-AED is the most effective inhibitor of tumour cell proliferation of the androstene steroids tested. In contrast, 17*β*-AED, which is chemically identical, only marginally inhibited proliferation at this concentration; therefore nonspecificity is not an issue. The IC_50_ for 17*α*-AED is <20 *μ*M for the glioblastoma and the lymphoma cells. This is particularly noteworthy when one considers that the IC_50_ of temozolomide on malignant glioma cell cultures has been reported to be 100 *μ*M or greater (Sankar *et al*, 1999; Kanzawa *et al*, 2003a, 2004). In addition, investigations by Yoshida *et al* (2003) on the antiproliferative action of DHEA on human cancer cell lines demonstrated that DHEA at 100 *μ*M could inhibit ∼50% of Hep G2 hepatoma cell proliferation and 30% of Caco-2 colonic adenocarcinoma cell proliferation; however, there was no significant reduction in tumour cell viability when DHEA was used at this dose. Also in this study, the investigators showed that etiocholanolone, a metabolite of DHEA; had the most potent cytotoxic activity, with an IC_50_ of ∼80 *μ*M on Hep G2 cells. This is approximately 10 times less potent than that of the cytotoxic effects of 17*α*-AED on human U937 lymphoma and T98G malignant glioma cells.

When used at high concentrations, 17*β*-AED, 7*α*-AET and 7*β*-AET were able to inhibit proliferation of the tumour cells, although the degree of inhibition was marginal as compared to that of 17*α*-AED. In terms of chemical structure, these results are interesting because 17*α*-AED and 17*β*-AED are chemically identical but differ in the placement, that is, above or below the plane of the androstene molecule, of a hydroxyl on the seventeenth carbon and yet exhibit an extreme difference their capacity to inhibit tumour cell proliferation. Furthermore, the addition of a hydroxyl group at the seventh position in either *α* or *β* orientation on the 17*β*-AED molecule does not increase the antiproliferative effects of 17*β*-AED. Overall, the results of the proliferation studies suggest that the potency of the antiproliferative effects of the androstenes may be dependent upon the placement and the number of the hydroxyl groups on the tetra-cyclic, androstene molecule.

Subsequent studies demonstrated that morphological effects could be noted on glioblastoma cultures after 18 h of exposure to 17*α*-AED, which included disruption of the cell monolayer and decreased adherence to the tissue culture plate. Also at this time point, reduced cell viability could be detected in the treated tumour cell cultures. The induction of cell death by 17*α*-AED were not as pronounced on U251MG cells, as compared with the T98G and U937 cells, suggesting that the U251MG cells may be more resistant to this neuro-steroid. Based on our previous work showing the effects of 17*α*-AED in human myeloid leukaemic cell lines, it is apparent that the effectiveness of this molecule is target cell dependent (Huynh and Loria, 1997). This is confirmed in this experiment.

Previous studies from our group have shown that 17*α*-AED treatment induces apoptosis in human breast and leukaemic cancer cells, as demonstrated by TUNEL and electron microscopy (Huynh and Loria, 1997; Huynh *et al*, 2000). However, it does not appear that apoptosis plays a major role in the death of malignant glioma cells treated with 17*α*-AED as shown by the nominal degree of DNA fragmentation; lack of caspase 3 cleavage and PARP cleavage as compared to sham-treated glioma cells. Additionally, preliminary electron microscopic analysis of the ultrastructure of T98G glioblastoma cells treated 3 days with 17*α*-AED did not reveal classical apoptotic features such as blebbing of the plasma membrane and DNA condensation (Graf *et al*, unpublished results). In some CNS diseases involving neuronal cell death, such as amyotrophic lateral sclerosis and Huntington's disease, traditional apoptotic features are not present (Dal Canto and Gurney, 1994; Turmaine *et al*, 2000).

Cytopathology revealed novel morphological changes in T98G cells treated for 30 h with 17*α*-AED. Most notable was the presence of numerous, large vacuoles in the cytoplasm of the treated cells, which was often accompanied with small, basophilic bodies. Acridine orange staining of 17*α*-AED-treated cells demonstrated that a large percentage of T98G glioma cells, but not U937 lymphoma cells, contain acidic vacuoles, and that their formation could be inhibited with bafilomycin A1.

Two forms of programmed cell death have been recently proposed (Bursch *et al*, 2000). Common features of type I programmed cell death, also referred to as apoptosis, include caspase activation, chromatin condensation and DNA cleavage (Jaattela, 2002). In contrast, autophagy is a form of type II programmed cell death, which is caspase independent, includes the formation of AVOs and may proceed in the absence of DNA cleavage (Lefranc and Kiss, 2006). Recent studies of human malignant glioma cells treated with temozolomide or arsenic trioxide have revealed that cell death occurs not by apoptosis, but rather by the induction of autophagy (Kanzawa *et al*, 2004, 2004). In our studies, human malignant glioma cells exposed to 17*α*-AED displayed features characteristic of autophagy, the presence of AVO and the ability of bafilomycin A1 to inhibit AVO formation, but not of apoptosis. Therefore, it is possible that the antiglioma effects of this neuro-steroid may be mediated by autophagic, type II programmed cell death. Biochemical and genetic studies are in progress to identify the extent to which 17*α*-AED induces autophagic cell death in the glioma cells, and to determine the molecular mechanisms involved. However, this is the first report of the antiglioma effects of 17*α*-AED.

To summarize, we have demonstrated that, of the androstene neuro-steroids tested, 17*α*-AED is the most potent inhibitor of human malignant glioma cells and lymphoma cells, with an IC_50_ in the rage of 10 to 20 *μ*M. In fact, 17*α*-AED used at a concentration of 25 *μ*M, can inhibit tumour cell proliferation by 80% or greater. These findings indicate that the cytotoxic effects of the neuro-steroids are dependent on the number and position of the hydroxyl groups on the androstene molecules as well as the aetiology of the tumour. It is apparent that the cytotoxic activity of 17*α*-AED on lymphoma cells is mediated by apoptosis; however, in malignant gliomas, it is likely that a nonapoptotic pathway of programmed cell death is utilized, such as autophagy. 17*α*-AED may have clinical relevancy in the treatment of brain tumour patients, because it is a neuro-steroid that can readily cross the blood brain barrier and possesses potent antiglioma activity.

## Figures and Tables

**Figure 1:**
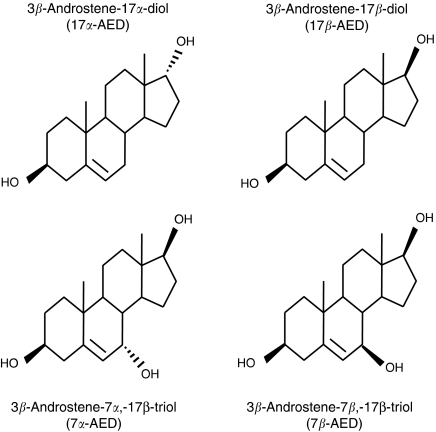
The molecular structure of 17*α*-AED, 17*β*-AED, 7*α*-AET and 7*β*-AET androstene neuro-steroids.

## References

[bib1] Ben Nathan D, Padgett DA, Loria RM (1999) Androstenediol and dehydroepiandrosterone protect mice against lethal bacterial infections and lipopolysaccharide toxicity. J Med Microbiol 48: 425–431 10229539 10.1099/00222615-48-5-425

[bib2] Boccuzzi G, Di Monaco M, Brignardello E, Leonardi L, Gatto V, Pizzini A, Gallo M (1993) Dehydroepiandrosterone antiestrogenic action through androgen receptor in MCF-7 human breast cancer cell line. Anticancer Res 13: 2267–2272 8297144

[bib3] Bursch W, Ellinger A, Gerner C, Frohwein U, Schulte-Hermann R (2000) Programmed cell death (PCD). Apoptosis, autophagic PCD, or others? Ann N Y Acad Sci 926: 1–12 10.1111/j.1749-6632.2000.tb05594.x 11193023

[bib4] Dal Canto MC, Gurney ME (1994) Development of central nervous system pathology in a murine transgenic model of human amyotrophic lateral sclerosis. Am J Pathol 145: 1271–1279 7992831 PMC1887498

[bib5] Huynh PN, Carter Jr WH, Loria RM (2000) 17 Alpha androstenediol inhibition of breast tumor cell proliferation in estrogen receptor-positive and -negative cell lines. Cancer Detect Prev 24: 435–444 11129985

[bib6] Huynh PN, Loria RM (1997) Contrasting effects of alpha- and beta-androstenediol on oncogenic myeloid cell lines *in vitro*. J Leukoc Biol 62: 258–267 9261340 10.1002/jlb.62.2.258

[bib7] Jaattela M (2002) Programmed cell death: many ways for cells to die decently. Ann Med 34: 480–488 12523503 10.1080/078538902321012423

[bib8] Kanzawa T, Germano IM, Komata T, Ito H, Kondo Y, Kondo S (2004) Role of autophagy in temozolomide-induced cytotoxicity for malignant glioma cells. Cell Death Differ 11: 448–457 14713959 10.1038/sj.cdd.4401359

[bib9] Kanzawa T, Germano IM, Kondo Y, Ito H, Kyo S, Kondo S (2003a) Inhibition of telomerase activity in malignant glioma cells correlates with their sensitivity to temozolomide. Br J Cancer 89: 922–929 12942127 10.1038/sj.bjc.6601193 PMC2394478

[bib10] Kanzawa T, Kondo Y, Ito H, Kondo S, Germano I (2003b) Induction of autophagic cell death in malignant glioma cells by arsenic trioxide. Cancer Res 63: 2103–2108 12727826

[bib11] Kemper EM, Boogerd W, Thuis I, Beijnen JH, van Tellingen O (2004) Modulation of the blood-brain barrier in oncology: therapeutic opportunities for the treatment of brain tumours? Cancer Treat Rev 30: 415–423 15245774 10.1016/j.ctrv.2004.04.001

[bib12] Lathe R (2002) Steroid and sterol 7-hydroxylation: ancient pathways. Steroids 67: 967–977 12398993 10.1016/s0039-128x(02)00044-2

[bib13] Lefranc F, Brotchi J, Kiss R (2005) Possible future issues in the treatment of glioblastomas: special emphasis on cell migration and the resistance of migrating glioblastoma cells to apoptosis. J Clin Oncol 23: 2411–2422 15800333 10.1200/JCO.2005.03.089

[bib14] Lefranc F, Kiss R (2006) Autophagy, the Trojan horse to combat glioblastomas. Neurosurg Focus 20: E7 10.3171/foc.2006.20.4.4 16709038

[bib15] Loria RM (1997) Antiglucocorticoid function of androstenetriol. Psychoneuroendocrinology 22(Suppl 1): S103–S108 9264155 10.1016/s0306-4530(97)00005-x

[bib16] Loria RM (2002) Immune up-regulation and tumor apoptosis by androstene steroids. Steroids 67: 953–966 12398992 10.1016/s0039-128x(02)00043-0

[bib17] Loria RM, Conrad DH, Huff T, Carter H, Ben Nathan D (2000) Androstenetriol and androstenediol. Protection against lethal radiation and restoration of immunity after radiation injury. Ann N Y Acad Sci 917: 860–867 11268417 10.1111/j.1749-6632.2000.tb05452.x

[bib18] Lubet RA, Gordon GB, Prough RA, Lei XD, You M, Wang Y, Grubbs CJ, Steele VE, Kelloff GJ, Thomas CF, Moon RD (1998) Modulation of methylnitrosourea-induced breast cancer in Sprague Dawley rats by dehydroepiandrosterone: dose-dependent inhibition, effects of limited exposure, effects on peroxisomal enzymes, and lack of effects on levels of Ha-Ras mutations. Cancer Res 58: 921–926 9500451

[bib19] Melvin WS, Boros LG, Muscarella P, Brandes JL, Johnson JA, Fisher WE, Schirmer WJ, Ellison EC (1997) Dehydroepiandrosterone-sulfate inhibits pancreatic carcinoma cell proliferation *in vitro* and *in vivo*. Surgery 121: 392–397 9122868 10.1016/s0039-6060(97)90308-1

[bib20] Nagata S, Nagase H, Kawane K, Mukae N, Fukuyama H (2003) Degradation of chromosomal DNA during apoptosis. Cell Death Differ 10: 108–116 12655299 10.1038/sj.cdd.4401161

[bib21] Nunez G, Benedict MA, Hu Y, Inohara N (1998) Caspases: the proteases of the apoptotic pathway. Oncogene 17: 3237–3245 9916986 10.1038/sj.onc.1202581

[bib22] Oliver FJ, de la RG, Rolli V, Ruiz-Ruiz MC, de Murcia G, Murcia JM (1998) Importance of poly(ADP-ribose) polymerase and its cleavage in apoptosis. Lesson from an uncleavable mutant. J Biol Chem 273: 33533–33539 9837934 10.1074/jbc.273.50.33533

[bib23] Padgett DA, Loria RM (1994) *In vitro* potentiation of lymphocyte activation by dehydroepiandrosterone, androstenediol, and androstenetriol. J Immunol 153: 1544–1552 8046232

[bib24] Padgett DA, Loria RM, Sheridan JF (2000) Steroid hormone regulation of antiviral immunity. Ann N Y Acad Sci 917: 935–943 11268423 10.1111/j.1749-6632.2000.tb05459.x

[bib25] Paglin S, Hollister T, Delohery T, Hackett N, McMahill M, Sphicas E, Domingo D, Yahalom J (2001) A novel response of cancer cells to radiation involves autophagy and formation of acidic vesicles. Cancer Res 61: 439–444 11212227

[bib26] Rao KV, Johnson WD, Bosland MC, Lubet RA, Steele VE, Kelloff GJ, McCormick DL (1999) Chemoprevention of rat prostate carcinogenesis by early and delayed administration of dehydroepiandrosterone. Cancer Res 59: 3084–3089 10397249

[bib27] Sankar A, Thomas DG, Darling JL (1999) Sensitivity of short-term cultures derived from human malignant glioma to the anti-cancer drug temozolomide. Anticancer Drugs 10: 179–185 10211548 10.1097/00001813-199902000-00006

[bib28] Schulz S, Klann RC, Schonfeld S, Nyce JW (1992) Mechanisms of cell growth inhibition and cell cycle arrest in human colonic adenocarcinoma cells by dehydroepiandrosterone: role of isoprenoid biosynthesis. Cancer Res 52: 1372–1376 1531325

[bib29] Simile M, Pascale RM, De Miglio MR, Nufris A, Daino L, Seddaiu MA, Muroni MR, Rao KN, Feo F (1995) Inhibition by dehydroepiandrosterone of growth and progression of persistent liver nodules in experimental rat liver carcinogenesis. Int J Cancer 62: 210–215 7622298 10.1002/ijc.2910620217

[bib30] Stupp R, Gander M, Leyvraz S, Newlands E (2001) Current and future developments in the use of temozolomide for the treatment of brain tumours. Lancet Oncol 2: 552–560 11905710 10.1016/S1470-2045(01)00489-2

[bib31] Stupp R, Mason WP, van den Bent MJ, Weller M, Fisher B, Taphoorn MJ, Belanger K, Brandes AA, Marosi C, Bogdahn U, Curschmann J, Janzer RC, Ludwin SK, Gorlia T, Allgeier A, Lacombe D, Cairncross JG, Eisenhauer E, Mirimanoff RO (2005a) Radiotherapy plus concomitant and adjuvant temozolomide for glioblastoma. N Engl J Med 352: 987–996 15758009 10.1056/NEJMoa043330

[bib32] Stupp R, Weber DC (2005b) The role of radio- and chemotherapy in glioblastoma. Onkologie 28: 315–317 15933418 10.1159/000085575

[bib33] Sundstrom C, Nilsson K (1976) Establishment and characterization of a human histiocytic lymphoma cell line (U-937). Int J Cancer 17: 565–577 178611 10.1002/ijc.2910170504

[bib34] Surawicz TS, Davis F, Freels S, Laws Jr ER, Menck HR (1998) Brain tumor survival: results from the National Cancer Data Base. J Neurooncol 40: 151–160 9892097 10.1023/a:1006091608586

[bib35] Traganos F, Darzynkiewicz Z (1994) Lysosomal proton pump activity: supravital cell staining with acridine orange differentiates leukocyte subpopulations. Methods Cell Biol 41: 185–194 7532261 10.1016/s0091-679x(08)61717-3

[bib36] Turmaine M, Raza A, Mahal A, Mangiarini L, Bates GP, Davies SW (2000) Nonapoptotic neurodegeneration in a transgenic mouse model of Huntington's disease. Proc Natl Acad Sci USA 97: 8093–8097 10869421 10.1073/pnas.110078997 PMC16675

[bib37] Walker MD, Green SB, Byar DP, Alexander Jr E, Batzdorf U, Brooks WH, Hunt WE, MacCarty CS, Mahaley Jr MS, Mealey Jr J, Owens G, Ransohoff J, Robertson JT, Shapiro WR, Smith Jr KR, Wilson CB, Strike TA (1980) Randomized comparisons of radiotherapy and nitrosoureas for the treatment of malignant glioma after surgery. N Engl J Med 303: 1323–1329 7001230 10.1056/NEJM198012043032303

[bib38] Yamamoto A, Tagawa Y, Yoshimori T, Moriyama Y, Masaki R, Tashiro Y (1998) Bafilomycin A1 prevents maturation of autophagic vacuoles by inhibiting fusion between autophagosomes and lysosomes in rat hepatoma cell line, H-4-II-E cells. Cell Struct Funct 23: 33–42 9639028 10.1247/csf.23.33

[bib39] Yoshida S, Honda A, Matsuzaki Y, Fukushima S, Tanaka N, Takagiwa A, Fujimoto Y, Miyazaki H, Salen G (2003) Anti-proliferative action of endogenous dehydroepiandrosterone metabolites on human cancer cell lines. Steroids 68: 73–83 12475725 10.1016/s0039-128x(02)00117-4

